# Lower lip squamous cell carcinoma: A Vietnamese case report of surgical treatment with reconstruction by local flap

**DOI:** 10.1016/j.ijscr.2018.11.025

**Published:** 2018-11-22

**Authors:** Hau Xuan Nguyen, Hung Van Nguyen, Hien Xuan Nguyen, Quang Van Le

**Affiliations:** a Department of Oncology, Hanoi Medical University, No 1 Ton That Tung Street, Dong Da District, Hanoi, Viet Nam; b Department of Oncology and Palliative Care, Hanoi Medical University Hospital, No 1 Ton That Tung Street, Dong Da District, Hanoi, Viet Nam; c Department of Head and Neck Surgery, Vietnam National Cancer Hospital, No 30 Cau Buou Street, Thanh Tri District, Hanoi, Viet Nam

**Keywords:** Lower lip cancer, Squamous cell carcinoma, Reconstruction, Local flap, Vietnam

## Abstract

**Introduction:**

Squamous cell carcinoma (SCC) of lower lip comprises over 25% of oral cancer. Surgical treatment plan for SCC patients includes tumor excision, lymph node dissection and reconstruction.

**Case presentation:**

A 68-year-old patient was admitted with 1-year history of tumor in his lower lip. Examination revealed a large lower lip tumor with expansion to the upper lip and a 2 cm submental lymph node. Biopsy result of the tumor was SCC and fine needle aspiration result of the lymph node was metastasis SCC. He underwent a complete removal of the lower lip and 1/3 external of the upper lip plus dissection of bilateral cervical lymph node plus reconstruction with local flap. After a 4-hour operation and 14-day hospitalization, patient recovered with flap in excellent condition and without any complications.

**Conclusion:**

Treatment with tumor excision, lymph node dissection and reconstruction by local flap is primary treatment for patients with lower lip cancer. Choice of reconstruction method depends on various factors, whereas V-Y advancement flap presents dominant advantage in lower lip cancer treatment.

## Highlights

• Many risk factors including age, sex, chronic exposure to solar radiation, tobacco, alcohol consumption, viral factors autoimmune diseases and using immunosuppressant drugs are associated with developing lip cancer.
• Treatment with tumor excision, lymph node dissection and reconstruction by local flap is primary treatment for patients with lower lip cancer.
• Bilateral V-Y advancement flap can be utilized in the reconstruction of lip cancer in case of large defect, as well as guarantee safety and adequate cosmetic and function for low-income patients.

## 1 Introduction

Head and neck cancer is the sixth most common type of cancer in the world [1]. Squamous cell carcinoma (SCC) of the lower lip comprises over 25% of oral cancer [2]. According to medical literature, lip SCC is more frequent in male patients aged over 45 years, those with chronic solar exposure, tobacco and alcohol drinking habits [2,3], and systematic lupus erythematosus [4,5].

SCC of lower lip can invade the deep muscle and mandible, as well as metastasize to neck lymph nodes [6]. Therefore, surgical management including tumor excision, lymph node dissection and reconstruction, plays an important role in the treatment plan [[7], [8], [9]]. The reconstruction of lip cancer includes primary closure, local flap and free flaps. Compared with free flaps, primary closure and local flap are advantageous because of less scar tissue after reconstruction, better cosmetic benefits, and not having the risk of donor site morbidity. Although primary closure is an easier surgical method and the scar has a linear shape, it might not be suitable for large resections in lip cancer surgery. In these cases, local flap, with various methods such as Abbe or Estlander flap, Bernard flap, and V-Y advancement flap, is a better choice for reconstruction [10].

In this report, we present a lower lip cancer case with large excision of both lips and simultaneous reconstruction using local flap. The work has been reported in line with SCARE criteria [11].

## 2 Case presentation

A male famer aged 68 years old was admitted to our institute for a tumor in his lower lip. This patient has a history of smoking (30 pack-year) and consuming alcohol for more than 30 years (approximately 200 ml per day). He was diagnosed as systemic lupus erythematosus (SLE) 20 years ago and had been treated with methylprednisolone for 17 years. For the last three years, his SLE status has been stable and thus, he discontinued the SLE treatment.

The patient reported that the tumor in the middle of his lower lip had appeared for a year and gradually increased in size without any pain or bleeding. He did not receive any treatment because of his financial issue. For the last few months, the tumor had rapidly grown, bled and become painful, so that he could not eat or clean his teeth.

**Fig. 1:**
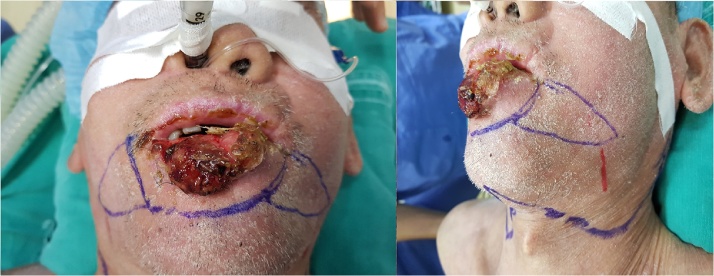
A 3 × 4 cm, raised, ulcerous, and bleeding tumor, developing in the lower lip and expanding to 1/3 external upper lip.

**Fig. 2:**
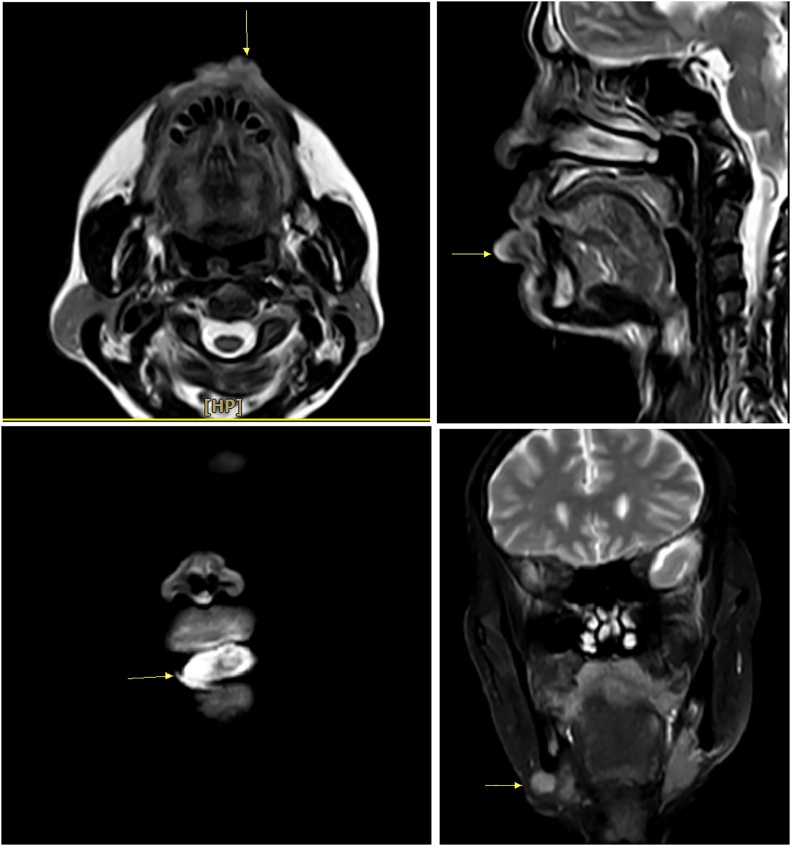
MRI Scan demonstrated a tumor in lower lip and a suspicious lymph node.

On examination, there was a 3 x 4 cm, raised, ulcerous, and bleeding tumor, developing in the lower lip and expanding to 1/3 external upper lip (Fig. 1). The submental lymph node was around 2 cm in diameter, firm, and hardly moveable. Ultrasound revealed a suspected metastatic submental lymph node with absent echogenic hilum. MRI Scan demonstrated a lesion in lower lip with size of 13 x 31 mm, which increased in T1W signal and T2W signal, strongly enhanced after contrast and did not invade surrounding tissue. A 2 cm and round lymph node was also identified (Fig. 2). The fine needle aspiration (FNA) result of the lymph node presented a metastatic squamous cell carcinoma and the biopsy result of the tumor confirmed squamous cell carcinoma (SCC). No abnormality was detected by a metastatic work-up. Therefore, the clinical staging of this patient was cT2N1M0. In addition, other para-clinical tests including full blood count, biochemistry profile, ds-DNA, ANA were normal, which indicated a stable status of SLE.

**Fig. 3:**
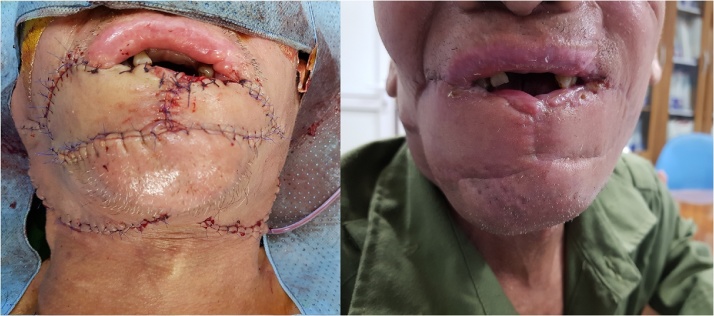
Patient after surgery (left picture: immediately after surgery, and right picture: 1 month after surgery).

**Fig. 4:**
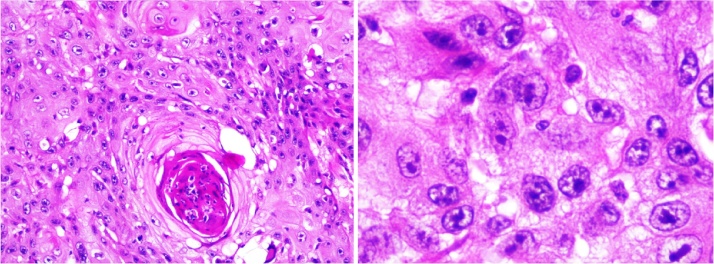
Final pathology result was squamous cell carcinoma.

The patient underwent surgery including a complete removal of the lower lip and 1/3 external of upper lip, and dissection of the bilateral cervical lymph nodes. The lower lip was reconstructed with V-Y advancement flap (Fig. 3). This operation, performed by a team of head and neck surgeons, was proceeded within 4 h. Patient was discharged after 14 days without any complications. Final pathology presented SCC (Fig. 4).

## 3 Discussion

Lip cancer can occur at any position along the upper or lower lip, but 90% of all cases are involved with the lower lip [12]. Lip cancer is a common malignancy of the head and neck region and accounts for about 12% of all cancers of this region as well as 25% of the cancers of the oral cavity [13]. There are many risk factors of developing lip cancer, including age (especially 60–70 years old), sex, chronic exposure to solar radiation, tobacco, alcohol consumption, viral factors (Human Papilloma Virus (HPV) 16 and 24, Herpes Virus (HSV) 1 and 2), autoimmune diseases and using immunosuppressant drugs [3,14,15]. In this case, the patient is a farmer and he often works in sunlight. He has also smoked and consumed a great deal of alcohol for a long duration. In addition, this patient has a history of systemic lupus erythematosus (SLE) for 20 years. The probability of SLE patients accompanied with cancer, mostly lymphoma, leukemia and lung cancer, is about 5% and the mean interval from SLE to the occurrence of the tumor is 13 years [16,17]. For SLE management, this patient was treated with immunosuppressant drugs in 17 years. All of these above factors may contribute to the increasing risk of lip cancer in this patient. On the other hand, the natural development of lip carcinoma is relatively slow, and median duration of symptom presentation was 9 months (range 1–95) prior to first examination [18]. In our report, the lesion in the lower lip appeared one year ago, but it did not affect his daily activities. Besides, he struggled with financial issue, so he refused to be admitted for any treatment. It cannot be denied that in Vietnam, household financial burden and poverty impacts of cancer treatment is substantial. Although financial protection is the most important aspect of health insurance coverage, in Vietnam, health insurance was not associated with statistically significant impacts on protecting households with cancer patients from impoverishment due to cancer treatment costs [19]. Consequently, many cancer patients cannot have access to and pursue continuously standard treatment.

Lip cancer remains one of the most curable malignancies in head and neck region since the 10-year survival rate can be as high as 98% and recurrence-free survival rate is more than 90% [6]. Surgery plays an important role in lip SCC management. The objectives of surgery are full-thickness resection, lymph node dissection and simultaneous reconstruction. Surgical methods depend mainly on the location and size of tumor. Lesions involving up to one third of the lower lip are usually treated by V-type or W-type excisions. Lesions involving between one and two thirds of the lower lip are commonly treated by regional flaps such as Abbe or Estlander flap. Larger lesions involving more than two thirds of the lower lip are treated with Bernard flap for middle lesions and nasolabial transpositional flap for lateral lesions, which borrow tissues from the cheek. If the defect is larger and the adjacent cheek tissue is inadequate, regional flap or free revascularized flap are utilized for reconstruction [8,20,21]. In this patient, we evaluated that his tumor involves >2/3 of the lower lip and invaded to 1/3 the right external upper lip, thus the choice of reconstruction could be either regional flap or revascularized flap. Although the revascularized flap provides high efficacy in not only cosmetic aspect but also lip function, its cost is as higher as 10 times than that of regional flap. This is the reason for our decision of performing regional flap reconstruction for this patient. After resecting the tumor with margins of 1.5 cm from the gross lesion, bilateral V-Y advancement flaps were used, in which the right V-Y advancement flap contents 1/3 right external upper lip. After reconstruction by bilateral V-Y advancement flaps, it is noticeable that this flap is able to reconstruct large defects and guarantee acceptable cosmetic and function quality (Fig. 3). As a result, bilateral V-Y advancement flap can be a suitable choice for selected patients.

## 4 Conclusion

The optimal treatment plan for patients with lower lip cancer includes tumor excision, lymph node dissection and reconstruction. Bilateral V-Y advancement flap can be utilized in the reconstruction of lip cancer in case of large defect, as well as guarantee safety and adequate cosmetic and function for low-income patients.

## Conflicts of interest

None.

## Sources of funding

None.

## Ethical approval

The study was approved by our research committee, Hanoi Medical University Hospital, Hanoi, Vietnam.

## Consent

The publication of this study has been consented by the relevant patient.

## Author contribution

Hau X. Nguyen: Main surgeon.

Hung V. Nguyen: Assistant surgeon, wrote manuscript.

Hien X. Nguyen: Assistant surgeon

Quang V. Le: Professor, revised manuscript.

## Registration of research studies

researchregistry4406.

## Guarantor

Quang V. Le, Professor, M.D, Ph.D.

## Provenance and peer review

Not commissioned externally peer reviewed.
